# Understanding treatment burden in hemophilia: development and validation of the Hemophilia Treatment Experience Measure (Hemo-TEM)

**DOI:** 10.1186/s41687-023-00550-6

**Published:** 2023-02-23

**Authors:** Meryl Brod, Donald M. Bushnell, Jesper Skov Neergaard, Laura Tesler Waldman, Anne Kirstine Busk

**Affiliations:** 1 The Brod Group, 219 Julia Ave, Mill Valley, CA USA; 2 Evidera | PPD, 7101 Wisconsin Ave, Bethesda, MD USA; 3 Novo Nordisk A/S, Vandtaarnsvej 112, Søborg Denmark

**Keywords:** Hemophilia, Patient-reported outcome measures, Concept elicitation, Interviews, Qualitative research, Psychometrics, Adult, Adolescent

## Abstract

**Background:**

To capture the broad range of treatment burden issues experienced by adolescent and adult people with hemophilia (PWH), the Hemophilia Treatment Experience Measure (Hemo-TEM) was developed. We describe the development of this new hemophilia-specific patient-reported outcome (PRO) measure including concept elicitation, cognitive debriefing, and psychometric validation.

**Results:**

Concept elicitation interviews were conducted with 5 clinical experts and 30 adult PWH in the United States (US). The qualitative analysis of these interviews and a review of the literature informed the PRO measure development. The project team reviewed concept endorsement rates and generated a 27-item preliminary version of the Hemo-TEM. Cognitive debriefing interviews were conducted to ensure participant understanding and item relevance in samples of (adolescent (n = 20) and adult (n = 14)) PWH in the US. The refined, validation-ready version of the Hemo-TEM included 30 items. Lastly, data from 3 clinical trials comprised the 4 analysis sets used for the psychometric validation with a sample size of N = 88. Item reduction dropped 4 items resulting in a final 26-item measure. Factor analysis generated 5 domains in the Hemo-TEM [injection difficulties (3 items), physical impact (6 items), treatment bother (7 items), interference with daily life (4 items), and emotional impact (6 items)] and a total score. All scores were reliable [internally consistent (0.84–0.88)]. For convergent validity, with the exception of one domain, all hypothesized associations were met. Preliminary sensitivity to change effect sizes were between − 0.30 and − 0.70. Meaningful change thresholds ranged from 6 points (physical impact and emotional impact) to 10 points (treatment bother) with 8 points for the Hemo-TEM total score.

**Conclusions:**

Findings from the concept elicitation, cognitive debriefing, and psychometric validation phases provide evidence that the Hemo-TEM is a well-designed, valid, and reliable measure of the burden of hemophilia treatment, including treatment impact on adolescent and adult PWH.

**Supplementary Information:**

The online version contains supplementary material available at 10.1186/s41687-023-00550-6.

## Background

Hemophilia is a recessive X-linked congenital bleeding disorder caused by a lack of clotting factor VIII (hemophilia A) or IX (hemophilia B) in the blood [1]. A chronic disease that requires a lifetime of monitoring and treatment, hemophilia is characterized by spontaneous and post-traumatic bleeding that is prolonged due to impaired clotting [2]. In addition to the pain and debilitation that can occur during bleeding episodes, its complications in joints and muscles can lead to chronic pain, severe joint damage, disability, and a dramatic impairment of health-related quality of life (HRQOL) [3, 4].

The current standard of care for hemophilia involves the venous infusion of clotting factor or bypassing agent to prevent or treat bleeding. Prophylaxis treatment reduces the number of joint bleeds, which in turn can prevent the development of arthropathy, the primary cause of morbidity and decreased quality of life (QOL) in people with hemophilia (PWH) [5]. In addition to the improvements in safety and efficacy, current treatments have made home infusion therapy possible [6, 7]. Despite being a welcome alternative to hospital or clinic-based treatments, home infusion can be a complicated and burdensome process, requiring phlebotomy skills, while the injections themselves may be painful and time-consuming to administer [8]. Venous access issues and other aspects of treatment burden, particularly the time required for an intravenous infusion, have been identified as major contributing factors to adherence issues including delayed treatment, not administrating treatment, or ceasing prophylactic treatment altogether [9, 10]. Currently available treatments for hemophilia have resulted in significant improvements in QOL for patients [11–15]. Given the on-going demands of monitoring and treating hemophilia, treatment burden is an important dimension to consider when evaluating treatment options. Unfortunately, there is limited published research on treatment burden in this population.

To capture the broad range of proximal treatment burden issues/impacts experienced by adolescent and adult PWH, the Hemophilia Treatment Experience Measure (Hemo-TEM) was developed. Condition-specific instruments, such as the Hemo-TEM, should have greater face validity, be more responsive to change over time than generic measures, and may be more useful to both clinicians and researchers to assess the burden of treatment for PWH. The purpose of this manuscript is to provide the development background for this newly developed hemophilia-specific patient-reported outcome (PRO) measure including concept elicitation (CE), cognitive debriefing (CD), and psychometric validation.

## Methods

The Hemo-TEM development and validation followed the process outlined in the FDA guidance for the development and validation of PRO measures [16, 17]. Institutional Review Board (IRB) approval was obtained for the development of the Hemo-TEM from Copernicus Group Independent Review Board®.

### Concept elicitation methodology

#### Literature review

PubMed (NLM); EMBASE, MEDLINE, and PsycINFO (ProQuest); and CINAHL (EBSCOhost) databases were searched to review published medical and psychological literature. The initial literature review was conducted November–December 2014 and was continuously updated based on search alerts throughout the research period, which ended in January 2021. The literature review provided the basis for developing the semi-structured interview guides that were used for the expert and PWH qualitative interviews.

#### Expert interviews

Clinical experts, based in the United States (US), were identified and recruited through a professional recruitment agency. A clinical expert was defined as a hematologist or nurse who currently had at least a 50% patient load in hemophilia and had been providing care to adult PWH for 5 or more years. Data from clinical experts were collected via 60-min telephone interviews which were recorded and transcribed verbatim. All interviews were conducted by trained, experienced qualitative researchers.

#### Interviews with adult PWH

Respondents were recruited from advocacy organizations and proprietary databases of hemophilia patients maintained by the authors as well as 2 professional recruitment vendors. Those recruited by the advocacy group were approached either by posting of the study flyer online or emailing members to inform them about the study and provide contact information for them to use if they were interested in participating. For the author’s proprietary database, potential participants were approached by email, and for the professional recruitment agency, their proprietary databases were used to email or telephone potential participants. Participants received an honorarium, approved by the ethics committee, for participating in the interviews.

Data from adult PWH were collected via telephone interviews. Four interviewers conducted the interviews. All interviewers were trained on interview procedures by the lead interviewer/analyst and met periodically to discuss findings and potential revisions needed to the discussion guide. Completed interviews were used to guide and inform subsequent interviews. If edits to the interview guide were needed, they were reviewed by the team to confirm appropriateness and wording before conducting the next interview.

Eligible participants had to:

- Be a male aged 18 years or older
- Be able to read, write and speak English
- Have a diagnosis of hemophilia A or B, with or without an inhibitor
- Have a factor level of < 2%
- Currently be receiving and self-administering either on demand or prophylactic treatment for hemophilia via factor replacement therapy or a bypassing agent
- Provide physician confirmation of diagnosis (As proof of diagnosis, participants were asked to provide proof of either a medical alert bracelet/necklace, medical alert card or medic alert form with diagnosis (hemophilia and type), prescription (prescription refill or photo/photocopy of prescription label) for an infusion agent specifically for hemophilia and/or a health care practitioner note or other communication.)

Participants were excluded if they had a cognitive impairment or any other medical condition which made them unable to participate in the interview.

The semi-structured interview guide was comprised of open-ended questions addressing the perceived burden of hemophilia treatment, and the specific variables that acted as moderators (i.e., factors that either increased or decreased the type or amount of burden experienced). The interviews were audio-recorded and transcribed verbatim. Informed consent was obtained from all participants.

### Qualitative data analysis

Data were analyzed using qualitative research methods based on an adapted grounded theory approach, entailing developing and refining a theory based on emerging concepts derived during the research process [18]. A preliminary code list was created based on the interview guides. Interviews were coded by the lead qualitative researcher and then reviewed by all interviewers and the scientific director of the project for agreement. Each transcript was skimmed once, coded, and reviewed multiple times. Transcripts were coded in chronological order, and emerging concepts were added to the coding scheme as they arose. Earlier transcripts were then re-evaluated for the new concepts. The codes were then organized into larger categories of major themes. The analysis was organized by the major themes and sub-themes that arose in the interviews. Interview transcripts were analyzed for content by theme using Dedoose [19], a qualitative analysis software program.

### Item generation

Data findings were reviewed and discussed by the entire project team (Interviewers, Scientific Director, and Qualitative Analysts) to revise/confirm codes as needed based on endorsement rates for concepts. Preliminary items for the measure were generated at the item generation meeting, using wording which reflected the wording used by respondents in the interviews as closely as possible. Item stems were chosen to most closely match the appropriate response options based on the content of the generated item.

### Cognitive debriefing

Participants were recruited from advocacy organizations and proprietary databases of hemophilia patients maintained by the authors as well as 2 professional recruitment vendors. The CD was conducted in 2 independent samples, unique from the concept elicitation sample (adolescent and adult), of PWH. The adult sample met the same eligibility criteria as the CE sample. The adolescent sample eligibility criteria were the same as for the adult sample eligibility criteria except adolescents had to be between the ages of 12 and < 18 years old and currently be self-administering their factor replacement therapy or bypassing agent the majority (> 50%) of the time. Interviews were conducted in blocks of 3 respondents. Edits were made in between blocks based on participant feedback.

The CD interviews were conducted according to a semi-structured telephone interview guide which was used to ask respondents questions employing a “think aloud” method, as well as verbal probing as needed [20]. An Item Definition List was prepared prior to the CD that defined the intended conceptual meaning of each item and instruction. Respondents were emailed an informed consent document and the Hemo-TEM prior to the interview and asked to print out and complete the measure 24–48 h before the interview.

During CD interviews, participants were asked to use their own words to indicate what they thought each item and instruction meant to them. Their responses were compared to the developed items’ and instructions’ intended meaning to confirm participant comprehension. The measure asks participants to think about their “current experience” to allow for some variability without the respondents being asked to recall their experience over an extended time period. The appropriateness and ability to answer for a recall period of their current experience were also examined in the CD interviews. Additionally, participants were asked whether the items were important or relevant to their experience with hemophilia and whether the items were in any way offensive or objectionable to them. Throughout and/or at the end of the interview, respondents were asked probed questions about response options and whether the formatting of the questionnaire was clear. Finally, comprehensiveness was assessed at the end of the interview, when respondents were asked whether there was anything not included that they would suggest be added.

### Psychometric validation methodology

The analyses used to evaluate Hemo-TEM item performance were in accordance with classical psychometric theory [21].

### Psychometric study sample

Data from 3 Novo Nordisk clinical trials [22, 23] with samples unique from the concept elicitation phase samples, comprised the 4 analysis sets which were used for the psychometric analyses.

#### Trial NN7170-4213 (alleviate 1) summary

The NN7170-4213 study was an international, multi-center phase 1 trial consisting of 2 parts: one single dose, dose escalation part (part A) and one multiple dose part (part B) with daily administrations of turoctocog alfa pegol (SC N8-GP) for a period of 3 months. The primary objective was to evaluate the safety of subcutaneous administration of SC N8-GP in patients with severe hemophilia A. The number of subjects who participated and were on trial product in part A was 24 and part B was 26.

*Major Inclusion Criteria*: 1) Male, age above or equal to 18 years at the time of signing informed consent (part A); 2) Male, age above or equal to 12 years at the time of signing informed consent (part B); 3) Diagnosis of congenital hemophilia A based on medical records (FVIII activity < 1%); 4) History of more than 150 exposure days to any FVIII containing products.

*Major Exclusion Criteria:* 1) Immune compromised patients due to human immunodeficiency virus (HIV) infection (defined as viral load ≥ 400,000 copies/mL and/or cluster of differentiation 4 + (CD4 +) lymphocyte count ≤ 200/µl performed at screening or defined by medical records no older than 6 months); 2) Any history of FVIII inhibitors (defined by medical records within 8 years of randomisation); 3) Inhibitors to FVIII (≥ 0.6 Bethesda units) at screening, measured by Nijmegen modified Bethesda method at central laboratory.

#### Trial NN7415-4255 (explorer5) summary

The NN7415-4255 study was a multi-center single arm trial, which aimed to assess the efficacy (primary objective) and safety of concizumab administered subcutaneously once daily to prevent bleeding episodes in patients with severe hemophilia A without inhibitors. Additionally, this trial aimed to assess the longer-term efficacy and safety of concizumab in patients with severe hemophilia A without inhibitors. The number of subjects who completed the trial was 32.

*Major Inclusion Criteria:* (1) Male patients aged ≥ 18 years at the time of signing informed consent, diagnosed with severe hemophilia A (FVIII activity < 1%), based on medical records or results at screening; (2) For patients being treated on-demand with FVIII replacement therapy, a minimum of six documented bleeding episodes during the 24 weeks (or twelve bleeds during 52 weeks) prior to screening.

*Major Exclusion Criteria*: (1) Known inherited or acquired bleeding disorder other than hemophilia A; (2) Major surgery conducted within one month prior to the initiation of trial activities or major surgery planned to occur during the trial; (3) Previous history of thromboembolic disease. Current clinical signs of thromboembolic disease, or patients who in the judgement of the investigator are considered at high risk of thromboembolic events; (4) Mental incapacity, unwillingness to cooperate or language barrier precluding adequate understanding and cooperation; (5) Patients who, at screening, have a significant infection or known systemic inflammatory condition which require systemic treatment according to the investigator’s judgement; (6) Hepatic dysfunction defined as elevated liver transaminases (ALT) > 3 times the upper limit of normal laboratory reference ranges at screening; (7) Renal impairment defined as estimated glomerular filtration rate (eGFR) ≤ 60 mL/min/1.73m^2^ based on serum creatinine measured at screening or evidence of renal damage; (8) Platelet count ≤ 100 × 10^9^/L at screening; (9) Fibrinogen level < the lower limit of normal at screening; (10) Presence of inhibitors (neutralising antibodies) to Factor VIII (≥ 0.6 Bethesda Units) at screening measured by the Nijmegen method; 11) History of inhibitors towards FVIII based on investigator’s knowledge or documentation in available medical records.

#### Trial NN7415-4310 (explorer4) summary

The NN7415-4310 study was a multi-center, randomised, open-label controlled trial to assess the efficacy (primary objective) of concizumab administered subcutaneously once daily to prevent bleeding episodes in hemophilia A and B patients with inhibitors. Additionally, this study aimed to assess the longer-term efficacy and safety of concizumab in hemophilia A and B patients with inhibitors and to establish the safety of treating breakthrough bleeding episodes with recombinant factor VIIa (rFVIIa) in these patients. The number of patients randomized was 26 and 25 completed the trial.

*Major Inclusion Criteria:* (1) Male hemophilia A or B patients with inhibitors aged ≥ 18 years at the time of signing informed consent; (2) Patients currently treated on-demand with a minimum of six bleeding episodes during the 24 weeks (or twelve bleeds during 52 weeks) prior to screening; (3) Documented history of high-titer inhibitors towards FVIII or FIX, defined as ≥ 5 Bethesda Units; (4) Patients currently in need of treatment with bypassing agents.

*Major Exclusion Criteria:* (1) Known inherited or acquired bleeding disorder other than hemophilia; (2) Major surgery conducted within one month prior to the initiation of trial activities or major surgery planned to occur during the trial; (3) Previous history of thromboembolic disease. Current clinical signs of thromboembolic disease or patients who in the judgement of the investigator are considered at high risk of thromboembolic events; (4) Mental incapacity, unwillingness to cooperate or language barrier precluding adequate understanding and cooperation; (5) Patients who, at screening, have a significant infection or known systemic inflammatory condition which requires systemic treatment according to the investigator’s judgement; (6) Hepatic dysfunction defined as elevated liver transaminases (ALT) > 3 times the upper limit of normal laboratory reference ranges at screening; (7) Renal impairment measured as estimated Glomerular Filtration Rate (eGFR) ≤ 60 ml/min/1.73m^2^ for serum creatinine measured at screening for patients without evidence of renal damage; (8) Platelet count ≤ 100 × 10^9^/L at screening; (9) Fibrinogen level < the lower limit of normal; (10) Ongoing or planned immune tolerance induction therapy or prophylaxis with FVIII or FIX; (11) Antithrombin levels below the normal reference range at screening.

### Analysis sets

#### Cross-sectional analysis set

The cross-sectional analysis set consisted of data for all subjects enrolled and completing the baseline assessments in NN7415-4255 and NN7415-4310. This sample was used for the primary psychometric analyses including, item reduction, factor analyses, internal consistency, and validity.

#### Retest analysis set

The retest analysis consisted of data for all subjects completing both the screening and the baseline assessment in NN7415-4255 and NN7415-4310. This was the sample used for the test–retest reproducibility analysis.

#### Sensitivity to change: analysis set

The longitudinal 24-week analysis set consisted of data for all subjects enrolled and completing the baseline and the 24-week follow-up assessments in NN7415-4255 and NN7415-4310. This was the sample used to explore 6-month sensitivity to change results.

#### Interpretability: meaningful change analysis set

The longitudinal analysis set consisted of data for all subjects who completed the baseline and each of the follow-up clinic assessments until a minimal improvement in treatment burden was reported in NN7415-4255, NN7415-4310, and NN7170-4213. This sample was used for the primary meaningful change analysis.

All statistical tests used a significance level of 0.05 (two-sided). Statistical tests involving multiple comparisons (e.g., analysis of variance (ANOVA) models with multiple groups) utilized Scheffe post-hoc tests, which adjusted for multiple comparisons and reduced the possibility of Type I error. Statistics were conducted using SPSS [24]. For convergent and discriminant hypotheses testing, when more than one hypothesis per subdomain was proposed, at minimum one had to be met to claim validity had been shown.

### Study measures

The validation battery included demographic questions; the Hemo-TEM; the Treatment Satisfaction Questionnaire for Medication (TSQM, version II), which assesses four key dimensions of treatment satisfaction: Effectiveness, Side Effects, Convenience, and Global Satisfaction [25]; the Sheehan Disability Scale, a 3-item measure developed to assess functional impairment in 3 inter-related domains: work/school, social, and family life [26]; the Validated Hemophilia Regimen Treatment Adherence Scale–Prophylaxis (VERITAS-Pro), a measure of treatment adherence [27]; the SF-36v2 PRO Health Survey, which consists of 8 components: physical functioning, bodily pain, general health perceptions, physical role functioning, emotional role functioning, social role functioning, vitality, and mental health [28]; the Self-Injection Assessment Questionnaire (SIAQ), which assesses overall experience with subcutaneous self-injection [29]; and the Patient Global Impression of Change (PGIC), an anchor measure of change in treatment burden since start of the clinical trial.

### Statistical analysis

#### Hemo-TEM descriptive characteristics

To assess item descriptive characteristics, missing data, ceiling effects, item-to-total correlations, and item-to-item correlations were examined. The item descriptive characteristics were then used to make item reduction decisions. Missing data was defined as any item which has no answer (is left blank). Any items having 5% or greater missing data were assessed for potential removal from the scale. To determine the ceiling effect, frequency statistics were run to count the number of “Never” or “Not at all” responses for each item. If the frequency of “Never” or “Not at all” responses was greater than 50%, the item was considered to have potential ceiling effect. Item-to-total correlations were examined between each item score and the total score. The item score is the individual score for each item in the measure and the total score is the summation of each item (exclusive of the item being examined) in its hypothesized subdomain. To calculate the item-to-total correlation, bivariate Pearson’s correlations for each item score against the total score were conducted. Any item with a value less than 0.40 indicated it may not be well associated with the rest of the items in the hypothesized scale. To determine the item-to-item correlation, reliability analysis for all item pairs were examined. Correlation coefficients greater than 0.70 were considered to be an indication of potential redundancy between the items. This threshold was based on the premise that a correlation of ≥ 0.70 is considered a strong association [30, 31]. However, the final decision to delete an item due to overlap with another item was based not only on the correlation but also took into consideration conceptual importance of the item.

#### Factor analyses

Exploratory factor analysis procedures were performed on the correlation matrices derived from the items comprising the Hemo-TEM. Varimax with Kaiser Normalization rotational methods were employed to achieve a meaningful set of factors. The appropriate number of factors to be extracted was determined as a function of the proportion of common variance accounted for, residuals analysis, scree plot examination, along with clinical and theoretical interpretability. Standardized factor loadings of at least 0.40 were considered acceptable [32].

A confirmatory factor analysis was also conducted to verify the final factor structure derived. The following fit indices were used to test and confirm the relationship between the observed variables and their underlying latent constructs: comparative fit index, goodness-of-fit index, and root mean square error of approximation.

#### Reliability

Cronbach’s alpha was used to assess internal consistency reliability [33, 34]. This statistic is used to analyze additive scales to determine to what degree the items within the scale are associated. A minimum correlation of 0.70 was the criteria for the instrument to be internally consistent.

Test–retest reliability, a measure of the stability of responses, was assessed using the intraclass correlation coefficient (ICC). A minimum coefficient of 0.70 was the criterion for the instrument to be considered to have adequate test–retest reliability.

#### Construct validity

Pearson’s correlations were computed to measure the association between the total score and subscale scores on the Hemo-TEM and the other measures included in the study. Convergent validity was considered to be supported if the Hemo-TEM scores were substantially correlated (≥ 0.40) with items or measures evaluating similar concepts. Specifically, the hypotheses using a two-tailed test at a p < 0.05 level were tested (Additional file 1: Table A).

Discriminant (known groups) validity was tested for the hypotheses using ANOVA tests at a p < 0.05 level) (Additional file 1: Table B).

#### Sensitivity to change

Distributional methods were applied using Cohen’s effect size (mean change in score divided by the standard deviation (SD) of the baseline score). A higher effect size indicates greater sensitivity to change. Standardized effect sizes of 0.2–0.5 were regarded as ‘small’, 0.5–0.8 as ‘moderate’ and those above 0.8 as ‘large’ [35].

#### Interpretability

##### Meaningful change thresholds

Change was examined with minimum differences in (total and domain scores) anchored to one-category-changes in the PGIC (e.g., severe to moderate) [36]. For changes reported in the PGIC, corresponding changes in the Hemo-TEM were evaluated for each PGIC change category; ‘A little better’ on the PGIC was hypothesized to be the meaningful change threshold [37].

## Results

### Concept elicitation results

#### Expert interviews

A total of 5 expert interviews were conducted. Practice settings included academic teaching hospitals (n = 4, 80%) and private group practice (n = 1, 20%). On average, experts had been treating PWH for 18.4 (range 9–30) years and spent 80% (range 50–90%) of their time in clinical practice, 13% (range 0–40%) teaching, and 7% (range 0–10%) in research activities.

Clinical experts identified the following burden of treatment issues:

- Overall Efficacy: Prophylaxis with factor products works very well, but the frequency of infusions may be burdensome for patients. While on demand regimens work, patients on these regimens with moderate and severe hemophilia are at risk for more bleeds and greater joint damage over time. Treatments for patients with inhibitors may be less effective.
- Impact of Disease Type: The burden of illness and treatment for patients with hemophilia A vs. hemophilia B are similar except that hemophilia B is often less severe. The burden of illness and treatment for patients with inhibitors tends to be greater than for those without inhibitors.
- Ease of Use/Injection Difficulties: PWH may experience several ease of use issues, including challenges with inserting the needle correctly, the frequency of their treatment regimen, the time to prepare and administer treatment, finding a good injection site/vein, and the infusion steps.
- Adherence: The majority of patients are adherent, but this may vary based on age, motivation, social support, the time it takes to self-treat, and the ease of accessing one’s veins. The key adherence issue is missing treatments, followed by delaying or postponing treatments, remembering to administer treatments, infusing per doctor recommendations/instructions, and recognizing when on demand treatment is needed.
- Emotional Burden: Patients may experience worry, frustration, and/or anger in relation to their treatment regimen, be in denial about or resistant to the need to adhere to their treatment regimen, or become “sick of” or fatigued by their regimen. The primary treatment-related sources of worry for patients are the risk of developing an inhibitor, the risk of acquiring an infection from their treatment, losing venous access, and their ability to self-infuse.
- Physical Burden: Patients may experience blown veins, pain, or bruising due to their treatment, and have difficulty accessing their veins. Patients who lose access to or have difficulty accessing their veins may need to use a port, catheter, or PICC line instead.
- Interference with Daily Life: Treatment regimens may negatively impact a patient’s ability to travel and interfere with their social activities.
- Treatment Satisfaction: Most patients appear to be satisfied with their treatment overall. The least liked aspects of current treatments are the intravenous mode of administration, time it takes to prepare and administer treatment, and frequency of treatment.

#### Interviews with PWH

##### Sample description

Thirty PWH across the US participated in the CE interviews, which lasted approximately one hour. All were male, and their average age was 38 (range 20–65) years. Thirty-five PWH (15 adults, 20 adolescents) participated in the CD interviews; however, one adult was excluded from the analysis as he gave conflicting responses to his hemophilia status and was not considered to be a reliable respondent. Participants in the final sample (n = 34) were male and their average age was 37 (range 20–64) years and 14 (range 12–17) years, for adults and adolescents, respectively.

Adult and adolescent CE and CD sample descriptions are listed in Tables 1 and 2.

**Table 1:** Concept elicitation and cognitive debriefing adult samples—summary

Concept elicitation interview respondent sample: summary				
Patient demographic characteristics	Prophylactic regimen (n = 19)	On demand regimen (n = 5)	Inhibitor regimen (n = 6)	Total (n = 30)
Age				
Mean	35.7	32.2	47.8	37.5
Median	35	31	53	35.5
(range)	(20–55)	(20–47)	(23–65)	(20–65)
Marital Status, n (%)				
Single	9 (47.4)	4 (80)	1 (16.7)	14 (46.7)
Married	6 (31.6)	1 (20)	4 (66.7)	11 (36.7)
Partnered	2 (10.5)	0 (0)	0 (0)	2 (6.7)
Separated	0 (0)	0 (0)	1 (16.7)	1 (3.3)
Divorced	2 (10.5)	0 (0)	0 (0)	2 (6.7)
Race/Ethnicity, n (%)				
Asian-American	1 (5.3)	1 (20)	0 (0)	2 (6.7)
Black/African-American	5 (26.3)	1 (20)	0 (0)	6 (20)
Latino/Hispanic	0 (0)	0 (0)	1 (16.7)	1 (3.3)
Mixed Race	1 (5.3)	1 (20)	1 (16.7)	3 (10)
White/Caucasian	11 (57.9)	2 (40)	4 (66.7)	17 (56.7)
Decline to answer	1 (5.3)	0 (0)	0 (0)	1 (3.3)
Work Status, n (%)				
Work part-time	6 (31.6)	0 (0)	1 (16.7)	7 (23.3)
Work full time	9 (47.4)	2 (40)	3 (50)	14 (46.7)
Student	1 (5.3)	1 (20)	0 (0)	2 (6.7)
Not working (disabled)	2 (10.5)	2 (40)	2 (33.3)	6 (20)
Not working	1 (5.3)	0 (0)	0 (0)	1 (3.3)
Education, n (%)				
High school	2 (10.5)	1 (20)	0 (0)	3 (10)
Vocational/technical	1 (5.3)	0 (0)	2 (33.3)	3 (10)
Some college	7 (36.8)	3 (60)	1 (16.7)	11 (36.7)
College degree	7 (36.8)	0 (0)	3 (50)	10 (33.3)
Graduate/professional	2 (10.5)	1 (20)	0 (0)	3 (10)

Patient general health, disease and treatment characteristics	Prophylactic regimen (n = 19)	On demand regimen (n = 5)	Inhibitor regimen (n = 6)	Total (n = 30)
Hemophilia Type, n (%)				
Type A	15 (78.9)	5 (100)	6 (100)	26 (86.7)
Type B	4 (21.1)	0 (0)	0 (0)	4 (13.3)
Hemophilia Severity Level, n (%)				
Severe	18 (94.7)	4 (80)	6 (100)	28 (93.3)
Moderate	1 (5.3)	1 (20)	0 (0)	2 (6.7)
Number of comorbidities				
Mean	3.3	1.8	2.5	2.9
Median	3	2	2.5	2.9
(range)	(1–7)	(0–4)	(0–4)	(0–7)
Lifetime Inhibitor Status, n (%)				
Current inhibitor			6 (100)	6 (20)
* … inhibitor status high*	–	–	4 (66.7)	4 (13.3)
* … inhibitor status low*	–	–	2 (33.3)	2 (6.7)
Past inhibitor	8 (42.1)	0 (0)	0 (0)	8 (26.7)
Never had inhibitor	11 (57.9)	5 (100)	0 (0)	16 (53.3)
Number of years with current inhibitor				
Mean	0 (0)	0 (0)	36.2^a^	
Median	0 (0)	0 (0)	37^a^	
(range)	0 (0)	0 (0)	(“several years”– 47)	
Current Regimen, n (%)				
Current Inhibitor regimen	–	–	6 (100)	6 (20)
On Demand regimen (no current inhibitor)	–	5 (100)	-	5 (16.7)
Prophylactic, regimen (no current inhibitor)	19 (100)	–	-	19 (63.3)

Cognitive debriefing respondent sample: summary				
Patient demographic characteristics	Prophylactic regimen (n = 10)	On demand regimen (n = 2)	Inhibitor regimen (n = 2)	Total (n = 14)
Age				
Mean	36.8	54.5	34.5	36.8
Median	29	54.5	34.5	29
(range)	(20–59)	(45–64)	(26–43)	(20–64)
Marital Status, n (%)				
Single	9 (90)	0 (0)	2 (100)	11 (78.6)
Married	1 (10)	2 (100)	0 (0)	3 (21.4)
Race/Ethnicity, n (%)				
Asian-American	2 (20)	0 (0)	0 (0)	2 (14.3)
Black/African-American	2 (20)	0 (0)	0 (0)	2 (14.3)
Latino/Hispanic	2 (20)	0 (0)	1 (50)	3 (21.4)
White/Caucasian	3 (30)	2 (100)	1 (50)	6 (42.9)
Decline to answer	1 (10)	0 (0)	0 (0)	1 (7.1)
Work Status, n (%)				
Work part-time	1 (10)	0 (0)	1 (50)	2 (14.3)
Work full time	4 (40)	1 (50)	1 (50)	6 (42.9)
Student	1 (10)	0 (0)	0 (0)	1 (7.1)
Not working (disabled)	2 (20)	1 (50)	0 (0)	3 (21.4)
Not working	2 (20)	0 (0)	0 (0)	2 (14.3)
Education, n (%)				
High school	3 (30)	0 (0)	0 (0)	3 (21.4)
Some college	4 (40)	1 (50)	1 (50)	6 (42.9)
College degree	2 (20)	1 (50)	0 (0)	3 (21.4)
Graduate/professional	1 (10)	0 (0)	1 (50)	2 (14.3)

Cognitive debriefing respondent sample: summary				
Patient general health, disease and treatment characteristics	Prophylactic regimen (n = 10)	On demand regimen (n = 2)	Inhibitor regimen (n = 2)	Total (n = 14)
Hemophilia Type, n (%)				
Type A	8 (80)	2 (100)	2 (100)	12 (85.7)
Type B	2 (20)	0 (0)	0 (0)	2 (14.3)
Hemophilia Severity Level, n (%)				
Severe	8 (80)	1 (50)	2 (100)	11 (78.6)
Moderate	2 (20)	1 (50)	0 (0)	3 (21.4)
Number of comorbidities				
Mean	2.5	4	1.5	2.6
Median	2	4	1.5	2
(range)	(0–6)	(3–5)	(1–2)	(0–6)
Lifetime Inhibitor Status, n (%)				
Current inhibitor			2 (100)	2 (14.3)
* … inhibitor status high*	–	–	2 (100)	2 (14.3)
Past inhibitor	2 (20)	0 (0)	0 (0)	2 (14.3)
Never had inhibitor	8 (80)	2 (100)	0 (0)	10 (71.4)
Current Regimen, n (%)				
Current Inhibitor regimen	-	-	2 (100)	2 (14.3)
On Demand regimen (no current inhibitor)	-	2 (100)	-	2 (14.3)
Prophylactic, regimen (no current inhibitor)	10 (100)	-	-	10 (71.4)

^a^n = 5, due to missing data

**Table 2:** Cognitive debriefing adolescent respondent sample: summary

Patient demographic characteristics	Prophylactic regimen (n = 18)	Inhibitor regimen (n = 2)	Total (n = 20)
Age			
Mean	14.4	12.5	14.3
Median	14	12.5	14
(range)	(12–17)	(12–13)	(12–17)
Race/Ethnicity, n (%)			
Asian-American	1 (5.6)	0 (0)	1 (5)
Black/African-American	2 (11.1)	0 (0)	2 (10)
Latino/Hispanic	1 (5.6)	0 (0)	1 (5)
White/Caucasian	13 (72.2)	2 (100)	15 (75)
Another Group Not Listed	1 (5.6)	0 (0)	1 (5)
Current School Grade, n (%)			
6	2 (11.1)	1 (50)	3 (15)
7	2 (11.1)	1 (50)	3 (15)
8	3 (16.7)	0 (0)	3 (15)
9	4 (22.2)	0 (0)	4 (20)
10	3 (16.7)	0 (0)	3 (15)
12	2 (11.1)	0 (0)	2 (10)
High school graduate/college	1 (5.6)	0 (0)	1 (5)
Another Group Not Listed	1 (5.6)	0 (0)	1 (5)

Patient general health, disease and treatment characteristics	Prophylactic regimen (n = 18)	Inhibitor regimen (n = 2)	Total (n = 20)
Hemophilia Type, n (%)			
Type A	17 (5.6)	2 (100)	19 (95)
Type B	1 (94.4)	0 (0)	1 (5)
Hemophilia Severity Level, n (%)			
Severe	18 (100)	2 (100)	20 (100)
Number of comorbidities			
Mean	.38	1	.45
Median	0	1	0
(range)	(0–2)	(1)	(0–2)
Lifetime Inhibitor Status, n (%)			
Current inhibitor	0 (0)	2 (100)	2 (10)
* … inhibitor status high*	-	1 (50)	1 (5)
Past inhibitor	3 (16.7)	0 (0)	3 (15)
Never had inhibitor	15 (83.3)	0 (0)	15 (75)
% of Time Self-Infuses Without Assistance			
Mean	86.3	96.5	87.3
Median	98	96.5	98
(range)	(50–100)	(94–99)	(50–100)
Current Regimen, n (%)			
Current Inhibitor regimen	–	2 (100)	2 (10)
Prophylactic, regimen (no current inhibitor)	18 (100)	–	18 (90)

##### Concept elicitation interviews

From the analysis of the CE interview transcripts of adult PWH, the following issues were identified:

- Overall Efficacy: The majority of participants considered their treatment regimen to be effective overall.
- Impact of Disease Type: No differences in the burden of treatment were identified based on hemophilia type (A versus B), though findings suggest that hemophilia with inhibitors is associated with a higher treatment burden. Overall, PWH treated with on demand therapy may experience less treatment burden in comparison with PWH on prophylaxis. Participants with current or past inhibitors generally concurred that having an inhibitor increased both the burden of illness and the burden of treatment. However, in many respects the experiences of PWH with inhibitors were comparable to those of PWH without inhibitors on prophylaxis. These findings suggest that while some aspects of treatment burden may be worse for PWH with inhibitors, they are not unique to this subpopulation.
- Ease of Use/Injection Difficulties: Most participants considered their treatment regimen to be convenient overall, particularly in comparison with previous treatments. The primary factors that continued to limit convenience were travel, venous access, treatment frequency, treatment time, treatment schedule, reconstituting medication, storage/refrigeration, treating outside one’s home, the infusion steps, packaging, and injecting.
- Adherence: Slightly over half of participants found it easy to adhere to their treatment regimen overall. The key adherence issues experienced included missing treatments, difficulty adhering to the treatment schedule, delaying treatments, refrigerating medication, taking treatments at the prescribed frequency, remembering to administer treatment, infusing in accordance with physician recommendations, and keeping track of and ordering medication and supplies.
- Emotional Burden: The primary emotional aspects of treatment reported by participants included worry, emotional resolve, nervousness/anxiety, relief/comfort, denial/resistance, frustration, a general positive emotional impact, feeling stressed or overwhelmed, and a wish to not have to infuse. Key treatment-related sources of worry included venous access issues, the risk of infection, the risk of developing an inhibitor, and the potential loss of venous access.
- Physical Burden: The primary issues affecting the physical burden of treatment were blown veins, pain, scarring, soreness, discomfort, and, for those who with a port, catheter or PICC line, infections.
- Interference with Daily Life: Participants reported that treatment interfered with travel and social activities, required them to stop activities to treat a bleed, and may have caused them to arrive at work late or leave work early.
- Economic Burden: Treatments and the majority of supplies were covered by insurance for study participants; therefore, the key treatment-related aspects of economic burden included co-pays or the need for supplemental insurance, issues related to insurance coverage, and the psychological burden of treatment costs.
- Treatment Satisfaction: Factors which effected satisfaction included mode of administration and treatment frequency, increased duration of product half-life, reconstitution step required, time required to prepare and administer treatment, and efficacy with respect to controlling bleeds. Additionally, participants cited other aspects including, convenience, safety, and the infusion volume.

Thematic saturation [18] was assessed for the 30 PWH interviews in the order in which they occurred. After the 16th interview, 75% of concepts had been discussed, and by the 26th interview, 95% of concepts were covered.

The top sub-concepts by Hemo-TEM domain and by treatment regimen are shown in Table 3.

**Table 3:** Top sub-concepts by Hemo-TEM domain

Sub-concept by Hemo-TEM domain	Prophylactic regimen (*n* = 19) *n* (%)	On demand regimen (*n* = 5) *n* (%)	Inhibitor regimen (*n* = 6) *n* (%)	Patient total (*n* = 30) *n* (%)
*Injection difficulties*				
Inserting the needle correctly	10 (53)	2 (40)	4 (67)	16 (53)
Fitting treatment into daily schedule	8 (42)	3 (60)	2 (33)	13 (43)
Treatment frequency	10 (53)	0	3 (50)	13 (43)
Time to prepare and administer treatment	9 (47)	1 (20)	3 (50)	13 (43)
Finding a good site/vein	7 (37)	1 (20)	1(17)	9 (30)
*Adherence Issues*				
Missing treatments	12 (63)	1 (20)	3 (50)	16 (53)
Delayed treatments	10 (53)	0	2 (33)	12 (40)
Refrigerating medication	8 (42)	0	2 (33)	10 (33)
Remembering to administer treatment	5 (26)	1 (20)	1 (17)	7 (23)
Infusing per doctor's recommendation/instructions	5 (26)	1 (20)	0	6 (20)
*Emotional burden of treatment*				
Worry	9 (47)	3 (60)	2 (33)	14 (47)
Emotionally resolved/used to treatment	5 (26)	2 (40)	4 (67)	11 (37)
Anxiety/nervousness	6 (32)	2 (40)	0	8 (27)
Relief/comfort	4 (21)	2 (40)	2 (33)	8 (27)
Denial/resistance	7 (37)	0	0	7 (23)
*Treatment-related physical burden*				
Blown veins	10 (53)	2 (40)	3 (50)	15 (50)
Pain	12 (63)	1 (20)	2 (33)	15 (50)
Scarring	9 (47)	2 (40)	3 (50)	14 (47)
Soreness	7 (37)	0	4 (67)	11 (37)
Coldness	7 (37)	2 (40)	1 (17)	10 (33)
*Interference with daily life*				
Travel	12 (63)	4 (80)	6 (100)	22 (73)
Miss a day of work	4 (21)	1 (20)	2 (33)	8 (27)
Social activities	6 (32)	0	1 (17)	7 (23)
Stopping activities to treat bleed	3 (16)	2 (40)	0	5 (17)
Leave work early/arrive late	4 (21)	0	1 (17)	5 (17)

### Item generation

Based on the qualitative analysis, a theoretical model of hemophilia treatment burden was developed. Domains were named to reflect the item content for that domain. Each domain was further broken down into subthemes, which were categorized as major, minor, or distal. The criteria for identifying whether the issues reported were considered as major were:

- Participant endorsement of at least 15%
- Considered as bothersome or important to participant
- Proximal to domain
- Specifically related to the burden of treatment (e.g., injection-related pain) rather than the burden of illness or illness progression (e.g., joint pain)

All issues that did not fulfill these criteria were categorized as minor, distal, or a modifier. For example, sensations of coldness or tingling during injection were not considered bothersome by participants and hence considered minor.

Based on this framework, potential items for the measure were generated for each major subtheme for each domain, using participants’ words as much as possible. A 5-level response scale was used in the measure; Dillman, et al., 2014 [38] generally recommends limiting scales to four or five categories as respondents can only hold a limited number of categories in their head at once. Too few response options (three or fewer) may lead to a significant reduction in both reliability and validity as respondents may not be able to accurately express subtle increments in severity or frequency of their response.

The preliminary version of the Hemo-TEM contained 27 items.

### Cognitive debriefing

For the adult sample (n = 14), a total of 5 blocks were necessary to refine the Hemo-TEM items in terms of readability and relevance. After block 5 interviews, all items and response options were found be comprehended well, the formatting and instructions were clear, and no additional concepts were identified for inclusion. All respondents understood the recall period and were able to answer in that timeframe.

As a result of the CD, the revised preliminary Hemo-TEM was comprised of 30 items arranged in 6 multi-part questions. This version was used for the adolescent debriefing.

For the adolescent sample (n = 20), a total of 5 blocks were necessary to refine the Hemo-TEM items in terms of readability and relevance. All items were reported to be important and relevant to the vast majority of participants, and they did not suggest any additional aspects of treatment burden to be included in the measure. Three-quarters (n = 15, 75%) reported that they had completed the entire measure on their own. Four participants (20%) reported seeking clarification from their parents on the instructions and/or specific items which were subsequently revised. All participants further reported that the amount of time it took to complete the measure was acceptable as was the formatting. Minor edits were made and the validation-ready version of the 30-item Hemo-TEM was generated.

### Psychometric validation results

#### Demographic characteristics

All participants (N = 88) were male and, on average, 35.9 years of age (SD 13.5, range 14–65). The majority were white (73.9%). The largest group of participants came from the US (12.5%) and the United Kingdom (11.3%).

#### Item characteristics

The item distribution and item-to-item correlations are shown below (Tables 4 and 5). For Item-to-total correlations, all items showed acceptable associations between each item against the rest of the items in its domain (excluding that item). All associations were above 0.52.

**Table 4:** Hemo-TEM item characteristics (total sample, n = 88)

	0-Not at all diff		1-A little diff		2-Somewhat diff		3-Very diff		4-Extremely diff	
	n	%	n	%	n	%	n	%	n	%
1a diff good place to inject	48	54.5%	25	28.4%	5	5.7%	6	6.8%	4	4.5%
1b diff put needle correctly	48	54.5%	23	26.1%	7	8.0%	5	5.7%	5	5.7%
1c diff find good place	32	36.4%	27	30.7%	17	19.3%	6	6.8%	6	6.8%
1d diff remember^a^	63	71.6%	22	25.0%	3	3.4%	0	0.0%	0	0.0%
1e diff exactly as instructed^a^	71	80.7%	13	14.8%	3	3.4%	1	1.1%	0	0.0%

	0-Never		1-Rarely		2-Sometimes		3-Often		4-Always	
	n	%	n	%	n	%	n	%	n	%
2a often postpone on purpose	45	51.1%	36	40.9%	6	6.8%	0	0.0%	1	1.1%
2b often postpone accident^a^	33	37.5%	42	47.7%	11	12.5%	1	1.1%	1	1.1%

	0-Never		1-Rarely		2-Sometimes		3-Often		4-Always	
	n	%	n	%	n	%	n	%	n	%
3a often soreness	43	48.9%	30	34.1%	10	11.4%	4	4.5%	1	1.1%
3b often physical discomfort	53	60.2%	21	23.9%	9	10.2%	4	4.5%	1	1.1%
3c often pain	50	56.8%	22	25.0%	11	12.5%	4	4.5%	1	1.1%
3d often bruising	30	34.1%	39	44.3%	13	14.8%	4	4.5%	2	2.3%
3e often ruptured veins	40	45.5%	27	30.7%	15	17.0%	5	5.7%	1	1.1%
3f often probs scarring	41	46.6%	20	22.7%	17	19.3%	6	6.8%	4	4.5%

	0-Not at all bothered		1-A little bothered		2-Somewhat bothered		3-Very bothered		4-Extremely bothered	
	n	%	n	%	n	%	n	%	n	%
4a bother number steps	40	45.5%	36	40.9%	9	10.2%	2	2.3%	1	1.1%
4b bother amt of time to prepare/give	40	45.5%	32	36.4%	13	14.8%	2	2.3%	1	1.1%
4c bother store meds	42	47.7%	28	31.8%	14	15.9%	2	2.3%	2	2.3%
4d bother carry meds	31	35.2%	30	34.1%	16	18.2%	8	9.1%	3	3.4%
4e bother often give treatment	34	38.6%	34	38.6%	14	15.9%	4	4.5%	2	2.3%
4f bother find time	43	48.9%	30	34.1%	10	11.4%	4	4.5%	1	1.1%

	0-Not at all interfering		1-A little interfering		2-Somewhat interfering		3-Very interfering		4-Extremely interfering	
	n	%	n	%	n	%	n	%	n	%
5a interfere travel/vacation	28	31.8%	31	35.2%	12	13.6%	11	12.5%	6	6.8%
5b interfere social acts	37	42.0%	38	43.2%	8	9.1%	3	3.4%	2	2.3%
5c interfere daily acts	41	46.6%	32	36.4%	11	12.5%	2	2.3%	2	2.3%
5d interfere work/school^b^	31	41.9%	33	44.6%	9	12.2%	0	0.0%	1	1.4%

	0-Never		1-Rarely		2-Sometimes		3-Often		4-Always	
	n	%	n	%	n	%	n	%	n	%
6a often anxious	47	53.4%	25	28.4%	13	14.8%	2	2.3%	1	1.1%
6b often frustrated	38	43.2%	32	36.4%	14	15.9%	3	3.4%	1	1.1%
6c often stressed	42	47.7%	28	31.8%	13	14.8%	3	3.4%	2	2.3%
6d often embarrassed^a^	55	62.5%	18	20.5%	7	8.0%	7	8.0%	1	1.1%
6e often worried infection	52	59.1%	17	19.3%	16	18.2%	2	2.3%	1	1.1%
6f often worried develop inhibitor	45	51.1%	22	25.0%	15	17.0%	4	4.5%	2	2.3%
6g often worried losing access vein	28	31.8%	23	26.1%	18	20.5%	13	14.8%	6	6.8%

^a^Item dropped after item reduction

^b^n=74 (n=12 reported “I do not currently work or go to school” and n=2 were missing data)

**Table 5:** Hemo-TEM item correlations (total population, n = 88)

	1a	1b	1c	1d	1e	2a	2b	3a	3b	3c	3d	3e	3f
1a diff good place to inject	1.000												
1b diff put needle correctly	.718^b^	1.000											
1c diff find good place	.553	.644	1.000										
1d diff remember^a^	.077	.242	.240	1.000									
1e diff exactly as instructed^a^	.242	.241	.390	.541	1.000								
2a often postpone on purpose	.117	.186	.279	.203	.300	1.000							
2b often postpone accident^a^	-.118	.091	.068	.381	.341	.363	1.000						
3a often soreness	.253	.153	.209	.090	.162	.230	.189	1.000					
3b often physical discomfort	.295	.245	.306	.036	.254	.304	.234	.683	1.000				
3c often pain	.270	.181	.233	.095	.200	.132	.290	.622	.685	1.000			
3d often bruising	.384	.282	.359	-.077	.046	.194	.110	.452	.387	.510	1.000		
3e often ruptured veins	.389	.310	.264	.082	.060	.100	-.028	.394	.364	.324	.412	1.000	
3f often probs scarring	.384	.303	.304	.117	.182	.089	.174	.513	.479	.524	.467	.469	1.000
4a bother number steps	.267	.209	.324	.186	.283	.323	.259	.238	.290	.288	.337	.205	.287
4b bother amt of time to prepare/give	.268	.259	.341	.265	.348	.409	.295	.196	.081	.201	.312	.146	.207
4c bother store meds	.231	.211	.475	.068	.171	.377	.165	.231	.063	.115	.329	.139	.282
4d bother carry meds	.294	.302	.485	.219	.275	.327	.233	.277	.161	.265	.420	.199	.349
4e bother often give treatment	.311	.270	.339	.234	.236	.461	.212	.240	.193	.263	.306	.246	.347
4f bother find time	.280	.284	.479	.277	.308	.431	.243	.269	.223	.228	.316	.250	.395
5a interfere travel/vacation	.442	.416	.545	.139	.270	.349	.129	.258	.253	.242	.485	.329	.383
5b interfere social acts	.277	.258	.463	.166	.311	.428	.241	.345	.393	.336	.377	.200	.330
5c interfere daily acts	.218	.146	.332	.253	.374	.325	.193	.361	.350	.242	.182	.175	.277
5d interfere work/school	.253	.138	.383	.190	.301	.340	.133	.443	.307	.257	.250	.350	.231
6a often anxious	.295	.297	.404	.173	.273	.313	.234	.344	.519	.363	.471	.375	.328
6b often frustrated	.363	.266	.349	.141	.269	.297	.214	.310	.384	.311	.464	.317	.444
6c often stressed	.196	.104	.248	.139	.342	.395	.137	.224	.385	.275	.431	.184	.224
6d often embarrassed^a^	.069	.138	.340	.302	.319	.307	.340	.228	.332	.273	.372	.291	.279
6e often worried infection	.247	.046	.183	.143	.242	.131	.153	.327	.446	.448	.230	.250	.300
6f often worried develop inhibitor	.141	.040	.236	-.063	.109	.147	.163	.469	.534	.485	.309	.276	.362
6g often worried losing access vein	.597	.595	.531	.025	.210	.164	.001	.386	.453	.419	.470	.613	.514

	4a	4b	4c	4d	4e	4f	5a	5b	5c	5d
4a bother number steps	1.000									
4b bother amt of time to prepare/give	.679	1.000								
4c bother store meds	.384	.527	1.000							
4d bother carry meds	.280	.511	.690	1.000						
4e bother often give treatment	.497	.578	.458	.544	1.000					
4f bother find time	.357	.519	.597	.719^b^	.653	1.000				
5a interfere travel/vacation	.385	.488	.627	.651	.550	.556	1.000			
5b interfere social acts	.513	.451	.457	.497	.606	.497	.690	1.000		
5c interfere daily acts	.410	.363	.325	.437	.507	.483	.587	.773^b^	1.000	
5d interfere work/school	.372	.283	.379	.434	.389	.590	.530	.629	.702^b^	1.000
6a often anxious	.488	.393	.406	.379	.453	.407	.506	.506	.421	.318
6b often frustrated	.432	.499	.436	.489	.517	.494	.600	.513	.459	.317
6c often stressed	.417	.448	.370	.392	.439	.432	.516	.558	.430	.269
6d often embarrassed^a^	.423	.370	.428	.465	.355	.442	.455	.499	.491	.314
6e often worried infection	.253	.090	.115	.210	.153	.146	.230	.222	.198	.240
6f often worried develop inhibitor	.195	.112	.195	.200	.142	.212	.296	.312	.210	.333
6g often worried losing access vein	.315	.331	.277	.363	.391	.262	.495	.413	.289	.293

	6a	6b	6c	6d	6e	6f	6g
6a often anxious	1.000						
6b often frustrated	.696	1.000					
6c often stressed	.666	.690	1.000				
6d often embarrassed	.647	.528	.576	1.000			
6e often worried infection	.369	.239	.283	.318	1.000		
6f often worried develop inhibitor	.347	.259	.314	.297	.617	1.000	
6g often worried losing access vein	.510	.468	.300	.247	.276	.393	1.000

^a^Item dropped after item reduction

^b^Very strong association (*r* > 0.70) between items

#### Item reduction

Based on item characteristics and conceptual importance, 4 items were dropped from the measure due to either high ceiling effects or high correlations with other proposed items. As 3 of these 4 items were in the hypothesized adherence domain and that adherence can be considered a distal consequence rather than a proximal impact of treatment burden, the concept was dropped in the resulting 26-item measure.

#### Factor analyses

An exploratory factor analysis (principal components analysis) was performed including the 26 items. As seen in Table 6, 5 factors presented: Injection Difficulties (3 items), Physical Impact (6 items), Treatment Bother (7 items), Interference with daily life (4 items), and Emotional Impact (6 items). When evaluating the items within each factor, there was predominant concordance with the original conceptual framework with the exception of one item. Item 6 g (*Because of taking your current treatment, how often do you feel worried about losing access to a vein*) factored into the Physical Impact domain, but, due to its concept of worry, it was determined to incorporate this item into the Emotional Impact domain.

**Table 6:** Hemo-TEM factor analysis (26 items)

		Factor				
		1	2	3	4	5
Injection difficulties	1a diff good place to inject	.821				
	1b diff put needle correctly	.811				
	1c diff find good place	.793				
Physical impact	3c often pain		.853			
	3b often physical discomfort		.819			
	3a often soreness		.749			
	3f often probs scarring		.710			
	3e often ruptured veins		.618			
	3d often bruising		.617			
Treatment bother	4b bother amt of time to prepare/give			.782		
	4c bother store meds			.718		
	4f bother find time			.700		
	4d bother carry meds			.672		
	2a often postpone on purpose			.623		
	4e bother often give treatment			.610		
	4a bother number steps			.559		
Interference	5c interfere daily acts				.743	
	5d interfere work/school				.717	
	5b interfere social acts				.577	
	5a interfere travel/vacation				.368	
Emotional impact	6a often anxious					.642
	6c often stressed					.627
	6b often frustrated					.621
	6e often worried infection					.577
	6f often worried develop inhibitor					.542
	6g often worried losing access vein		.602			.211

A post-hoc confirmatory factor analysis was also performed using IBM® SPSS® Amos™ [39, 40]. Adequate fit indices were seen: comparative fit index (0.988), goodness-of-fit index (0.993, adjusted 0.988), and root mean square error of approximation (0.06) [41, 42].

Additionally, a higher order factor analysis was conducted on the 5 subscale scores to determine the ability to create an overall score of treatment burden. The subscales factored into a single component with 64.1% of total variance explained.

#### Reliability

Internal consistency reliability for the Hemo-TEM was examined using Cronbach’s alpha. All coefficients exceed the threshold of 0.70 indicating internally consistent scales and adequate test–retest reliability (see Table 7). Test–retest reliability was also found to be adequate with all but one domain and the overall score exceeding the 0.70 threshold. For that domain, the range included the 0.70 threshold.

**Table 7:** Evidence for internal consistency of the Hemo-TEM

	Cronbach’s alpha by domain	Test–Retest ICC (C.I.)
Injection difficulties	0.863	0.826 (0.745–0.882)
Physical impact	0.876	0.827 (0.747–0.883)
Treatment bother	0.879	0.824 (0.743–0.882)
Interference	0.869	0.625 (0.476–0.738)
Emotional impact	0.843	0.791 (0.697–0.858)
Overall	0.949	0.882 (0.826–0.921)

#### Construct validity

Construct validity of the Hemo-TEM was found to be acceptable. For convergent validity, with the exception of one domain, all hypothesized associations were met: the Injection Difficulties domain had a lower-than-expected correlation with the TSQM Convenience domain. However, the Hemo-TEM Interference domain did have a strong correlation (r = – 0.63) with the TSQM Convenience domain. Additionally, a post-hoc analysis was performed using the Hemo-TEM Treatment Bother domain and the TSQM Convenience domain, which found these 2 domains were strongly correlated (r = – 0.67) (see Table 8). For Known-groups validity, all hypotheses for the retained domains were met except that analysis of one hypothesis was not testable due to the sample composition.

**Table 8:** Evidence for convergent validity of the Hemo-TEM

Domain	Measure	Relationship or hypothesis	Correlation coefficient
Hemo-TEM total score	TQSM Total Score	Moderate to strong correlation	– 0.55
Hemo-TEM injection difficulties	TQSM Convenience	Moderate to strong correlation	– 0.19
Hemo-TEM physical impact	SIAQ Pain + Skin Reaction	Moderate to strong correlation	0.68
	Post-hoc: Sheehan Disability Scale	Moderate to strong correlation	0.45
Hemo-TEM Interference	Sheehan Disability Scale	Moderate to strong correlation	0.57
	Post-hoc: TSQM Convenience	Moderate to strong correlation	– 0.63
Hemo-TEM emotional impact	SF-36 Mental Health	Moderate to strong correlation	– 0.47
Hemo-TEM treatment bother	TSQM Convenience	Moderate to strong correlation	– 0.67

#### Sensitivity to change

Table 9 shows the results of the change over time of the participants who completed a follow-up assessment 24 weeks post baseline. Marked improvements were noted for all Hemo-TEM domains (ranging between 6.1 and 14.0 points on a 0–100-point scale). Associated effect sizes (mean change divided by the baseline standard deviation) ranged from – 0.30 (Physical Impact) to – 0.70 (Interference), indicating that the Hemo-TEM is sensitive to change.

**Table 9:** Sensitivity to change

	Baseline			WEEK 24			Mean difference	Effect size^a^
	N	Mean	SD	N	Mean	SD		
Injection difficulties (3 items)	59	27.1	28.5	59	14.1	23.2	– 13.0	– 0.46
Physical impact (6 items)	59	21.6	20.7	59	15.5	19.1	– 6.1	– 0.30
Treatment bother (7 items)	59	22.3	19.0	59	10.7	14.6	– 11.7	– 0.62
Interference (4 items)	59	23.0	20.0	59	9.0	16.9	– 14.0	– 0.70
Emotional impact (6 items)	59	22.5	18.4	59	12.1	14.7	– 10.4	– 0.56
Total score	59	23.3	16.8	59	12.3	15.6	– 11.0	– 0.66

^a^Effect size: mean change score divided by the standard deviation of the baseline score

#### Interpretability

##### Meaningful change thresholds

For this study, change was monitored over time aiming to capture Hemo-TEM change when participants experienced initial improvements as reported by 2 PGIC questions: 1) change before you started this study; and 2) change when you first started giving yourself injections as part of this study. Meaningful change thresholds for the Hemo-TEM were derived from associated change in Hemo-TEM scores for those patients that had responded “A Little/Somewhat” in either of the 2 PGIC questions as shown in Table 10.

**Table 10:** Meaningful change of the Hemo-TEM

	Hemo-TEM Mean (SD)					
	Injection difficulties	Physical impact	Treatment bother	Interference	Emotional impact	Total score
PGIC: Compared to the hemophilia treatment you were taking BEFORE you started this study, would you say your current experience with taking your treatment is						
A Little/Somewhat (n = 12)	– 10.4 (31.2)	– 0.4 (13.9)	– 8.9 (22.7)	– 6.3 (10.0)	– 4.5 (16.5)	– 6.1 (13.3)
A Good Deal (n = 9)	– 13.0 (24.0)	– 8.8 (17.4)	– 12.3 (13.7)	– 4.2 (17.1)	– 8.8 (10.9)	– 9.4 (10.6)
A Great Deal (n = 19)	– 13.6 (17.6)	– 8.8 (12.2)	– 10.3 (11.8)	– 9.5 (12.4)	– 7.0 (7.6)	– 9.9 (7.8)
A Very Great Deal (n = 15)	– 27.8 (37.2)	– 10.0 (17.9)	– 17.9 (21.4)	– 21.3 (20.2)	– 13.9 (18.3)	– 18.2 (19.8)
Total (n = 55)	– 16.7 (28.2)	– 7.3 (15.2)	– 12.4 (17.6)	– 11.1 (16.2)	– 8.6 (13.9)	– 11.2 (14.0)
PGIC: Compared to when you first started giving yourself injections as part of this study, would you say your experience with giving yourself injections is						
A Little Worse (n = 1)	8.3 (–)	12.5 (–)	– 3.6 (–)	0.0 (–)	– 4.2 (–)	2.6 (–)
A Little/Somewhat (n = 11)	– 9.1 (28.2)	– 6.1 (10.6)	– 14.3 (21.4)	– 8.5 (14.3)	– 7.2 (17.4)	– 11.6 (12.3)
A Good Deal (n = 11)	– 12.9 (21.5)	– 7.6 (15.8)	– 15.3 (12.9)	– 6.3 (15.6)	– 7.6 (9.8)	– 9.9 (9.6)
A Great Deal (n = 15)	– 15.0 (32.0)	– 8.6 (12.1)	– 13.1 (20.1)	– 13.8 (16.9)	– 8.9 (9.2)	– 11.9 (14.5)
A Very Great Deal (n = 10)	– 21.7 (31.5)	– 10.8 (20.8)	– 14.3 (16.1)	– 19.4 (21.3)	– 14.6 (22.0)	– 16.1 (20.2)
Total (n = 48)	– 17.0 (28.7)	– 7.8 (14.7)	– 13.9 (17.5)	– 11.7 (17.1)	– 9.3 (14.5)	– 11.9 (14.2)

Taking into consideration both PGIC results above, and given the variance in each analysis, the meaningful change thresholds for the Hemo-TEM are 9.5 points for Injection Difficulties (between 9.1 and 10.4 points), 6 points for Physical Impact (putting more value on the second PGIC item as 0.4 points may be too minimal), 10 points for Treatment Bother (between 8.9 and 14.3), 7 points for Interference (between 6.3 and 8.5), 6 points for Emotional Impact (between 4.5 and 7.2), and 8 points for the Hemo-TEM total score (between 6.1 and 11.6).

### Final measure

**Fig. 1:**
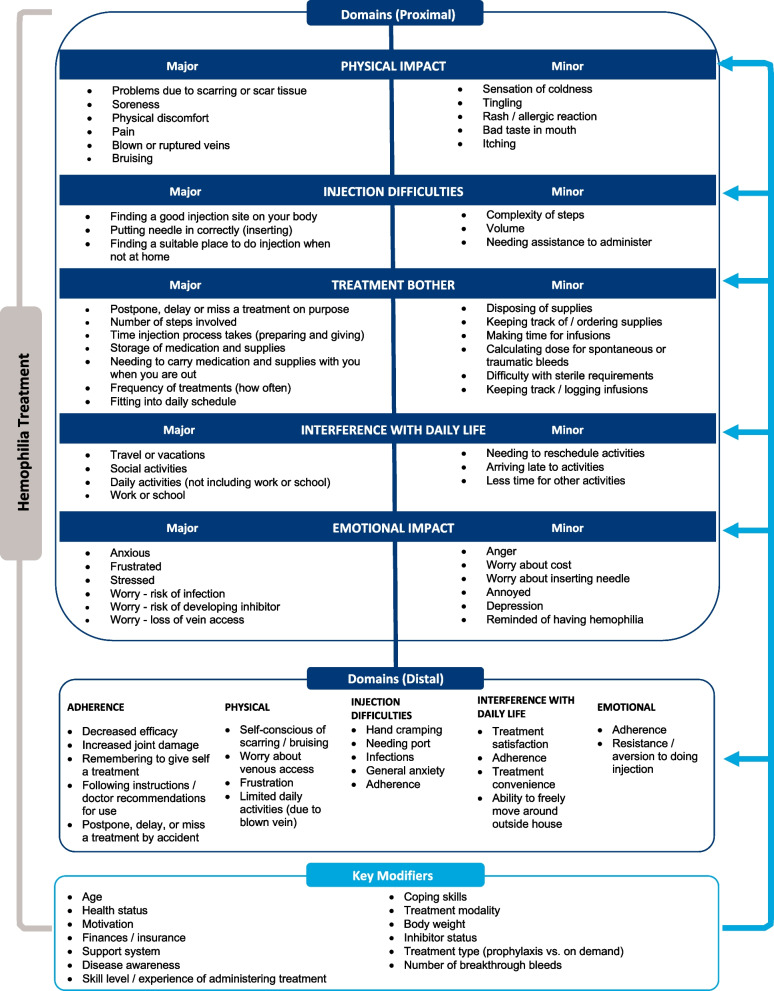
Theoretical Model

**Fig. 2:**
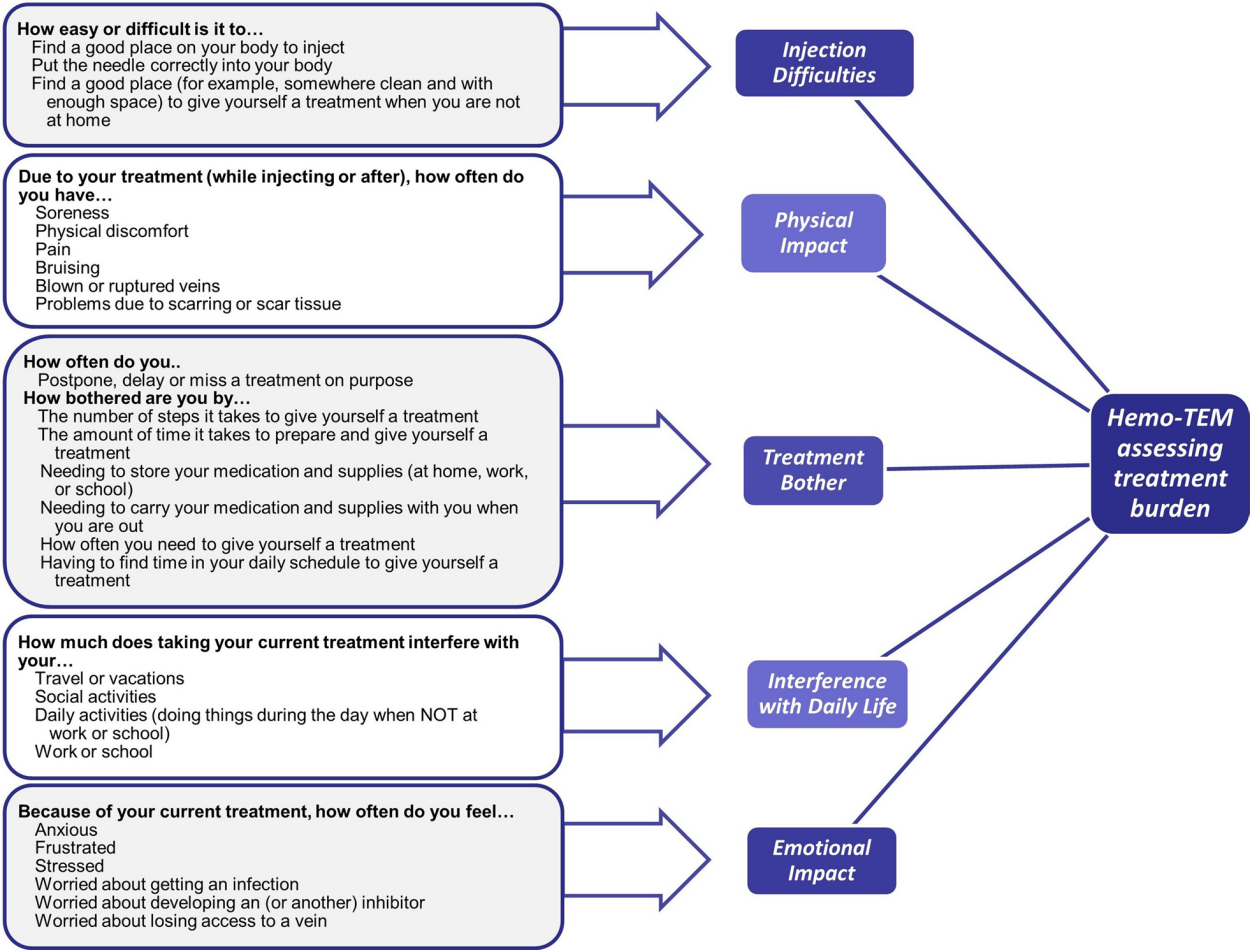
Hemo-TEM Conceptual Framework

Based on the data collected in the CE and the validation phases of the Hemo-TEM measure development, the 26-item measure was finalized based on the theoretical model (Fig. 1). The measure has 6 item stems, followed by items whose response categories match the framing of the item stem. Instructions for completing the measure precede the first item stem. Figure 2 shows the final structure of the Hemo-TEM.

### Scoring of the Hemo-TEM

The Hemo-TEM consists of 26 items covering five domains: Injection Difficulties, Physical Impact, Treatment Bother, Interference, and Emotional Impact. Prior qualitative evidence and the quantitative evidence reported here were used collectively to inform the provisional scoring algorithm. In an effort to provide an easy to score and an easy to interpret measure, none of the items should be weighted more heavily in their respective subscale score than the others. For each domain, a single score is created by summing the domain items and converting onto a 0–100-point scale. All domain scores are transformed onto a 0–100-point scale with higher scores indicating greater burden.

## Discussion

Findings from the CE, CD, and psychometric validation phases provide evidence that the Hemo-TEM is a well-designed, valid, and reliable measure of the burden of hemophilia treatment, including treatment impact on adolescent and adult PWH. Although other measures exist which may capture some aspects of treatment burden [43], the Hemo-TEM is the first measure designed specifically to only assess the broad spectrum of treatment burdens distinct from disease burden or treatment satisfaction.

Adequate assessment of treatment burden needs to capture its multidimensionality. The Hemo-TEM, with domains of physical impact, injection difficulties, interference in daily life, emotional impact, and treatment bother, can be considered a multidimensional measure, which includes the domains important to PWH found in the literature and as identified in the CE interviews lead to better outcomes. It has been shown that aspects and challenges of treatment may have significant impacts on QOL. For example, reductions in frequency of bleeding episodes, have been shown to improve QOL [44, 45]. Additionally, reduced infusion frequency and longer duration of factor coverage result in multiple advantages such as greater ability to participate in physical activities, less time to schedule and administer treatment, and improved emotional health [46]. Treatments with less invasive routes of administration or frequency of dosing may increase adherence and improve outcomes [47]. The Hemo-TEM, as a multidimensional measure, will allow for more targeted and direct investigations of treatment burden as it relates to outcomes such as HRQOL and adherence.

Similarities between clinician and patient participant CE interviews should be noted. Across domains, the key drivers of treatment burden cited by both clinicians and PWH were venous access issues, treatment frequency, and the time required to prepare and administer treatment. Understanding these key drivers and the patient experience with treatment enable us to better tailor treatments to improve the patient experience and effectiveness of interventions. Treatments offering benefits in these areas, such as less frequency, should lessen treatment burden for PWH, thus reducing both proximal and distal consequences of treatment burden.

The concept of adherence was not included in the final Hemo-TEM, as it did not perform psychometrically and conceptually, one could view adherence as a more distal consequence rather than proximal impact of treatment burden and thus not suitable for a measure of the proximal impacts designed to capture treatment benefit in a clinical trial setting. Further, it has been suggested that quantitative methods of assessing adherence, such as counting of factor concentrate vials returned, may be a more accurate methodology for the assessment of adherence [48]. Thus, we suggest that given the importance of adherence in treatment efficacy, adherence be assessed along with treatment burden [49–51], i.e., alongside the Hemo-TEM rather than as part of it. Whichever side of these discussions one leans towards, it seems evident that reduced treatment burden would result in greater treatment adherence [52].

As with any study, there are limitations to this one. Although we did find that our measure was responsive to change based on the patient-reported anchors, additional research is needed to confirm our preliminary estimates of meaningful change. Also, it would be interesting to investigate responsiveness to clinical endpoints such as a clinician rated improvement assessment, hemophilia clinical endpoints such as number of bleeds, or biomarkers. However, it should be noted that clinical endpoints do not necessarily correlate well with the patient-reported outcomes and should be considered supportive rather than substitutive for the patient perspective [53, 54]. The qualitative study was conducted only in the US as was the measure’s validation in a clinical trial, which may limit its generalizability to other Western and non-Western countries. The sample characteristics may have been influenced by restraints of the trials' eligibility criteria and methodology. Additionally, the measure was developed to be appropriate for both adolescents and adults which required that certain items and concepts be framed in a way to be appropriate to both age groups. Therefore, nuances to certain concepts, although included in the higher-level concept, were not specified. For example, certain aspects of social life, such as dating, are often more dependent on age, marital status, or personal interest. As validation is an iterative process, further qualitative CE in non-US, “real world” populations would be illustrative and increase our understanding of treatment burden for this population.

Treatment burden may be a primary reason that PWH change treatments [55]. For example, the need to reduce treatment burden by reducing the number of injections, has led to the development of newer treatments [56]. Adequately assessing treatment burden, in all its complexity, is necessary to allow clinicians to use this information to better tailor treatments to a patient’s needs. This should lead to better treatment outcomes and improved HRQOL for PWH.

## Conclusions

Assessment of the burden of hemophilia treatment is critical to informing our understanding of its impact on the disease and patient’s lives from both the research and the clinical perspective. The qualitative study supports the appropriateness and comprehensiveness of the Hemo-TEM to assess the intended concept of treatment burden in PWH, and the psychometric analyses confirmed that the Hemo-TEM has adequate measurement properties. The Hemo-TEM should prove useful in clinical trials to assess hemophilia treatment burden, and to clinicians in tailoring treatments to patient characteristics and situations. With this understanding, more efficacious treatments with better, patient-centered treatment outcomes beyond clinical efficacy can evolve.

## Supplementary Information

**Additional file 1**. Construct Validity and Known Groups Validity Hypotheses.

## Data Availability

The data for the research presented in the publication may be available on a case-by-case basis for reasonable requests from the corresponding author.

## Abbreviations

ANOVA: Analysis of variance
CD: Cognitive debriefing
CE: Concept elicitation
Hemo-TEM: Hemophilia Treatment Experience Measure
ICC: Intraclass correlation coefficient
IRB: Institutional review board
HRQOL: Health related quality of life
PGIC: Patient Global Impression of Change
PWH: People with hemophilia
PRO: Patient-reported outcome
SD: Standard deviation
SIAQ: Self-Injection Assessment Questionnaire
TSQM: Treatment Satisfaction Questionnaire for Medication
QOL: Quality of life
US: United States
VERITAS-Pro: Validated Hemophilia Regimen Treatment Adherence Scale–Prophylaxis
