# Supplementary material for: Understanding treatment burden in hemophilia: development and validation of the Hemophilia Treatment Experience Measure (Hemo-TEM)
Source: J Patient Rep Outcomes. 2023 Feb 23;7:17. doi: 10.1186/s41687-023-00550-6 (PMC9950311; doi:10.1186/s41687-023-00550-6)
Supplement: Supplementary file 1 — Additional file 1. Construct Validity and Known Groups Validity Hypotheses. [file 41687_2023_550_MOESM1_ESM.docx]

Understanding Treatment Burden in Haemophilia: Development and Validation of the Hemophilia Treatment Experience Measure (Hemo-TEM)

# Supplementary Material

**Table A. Construct Validity Hypotheses**

| 1: | The Hemo-TEM total score will be related to the TQSM total score |
| --- | --- |
| 2: | The Hemo-TEM Injection Difficulties domain will be related to the TQSM Convenience domain |
| 3: | The Hemo-TEM Physical Impact domain will be related to the SIAQ pain and skin reactions during or after the injection domain |
| 4: | The Hemo-TEM Interference domain will be related to the Sheehan Disability Scale total score |
| 5: | The Hemo-TEM Emotional Impact domain will be related to the SF-36 Mental Health domain |

**Table B. Known Groups Validity Hypotheses**

| 6: | Those who need more time to prepare and administer their treatment will report greater burden on the total score of the Hemo-TEM |
| --- | --- |
| 7: | Those who used prophylaxis as their primary treatment before entering the study will report experiencing less difficulty on the Hemo-TEM Injection Difficulties domain as compared to those who used on-demand treatment |
| 8: | Those who report having a busier and more stressed day will report greater interference on the Hemo-TEM Interference domain |
| 9: | People who are older will report a greater emotional impact in the Hemo-TEM Emotional Impact domain as compared to those who are younger |
